# Disease Priorities in Zambia

**DOI:** 10.4269/ajtmh.16-0130a

**Published:** 2016-07-06

**Authors:** Duncan Mara

**Affiliations:** ^1^ School of Civil Engineering, University of Leeds, Leeds, United Kingdom. E-mail: d.d.mara@leeds.ac.uk.

Dear Sir:

I read with great interest the article by Ng and others entitled “Assessing the contribution of malaria vector control and other maternal and child health interventions in reducing all-cause under-five mortality in Zambia.”^1^ Considering that malaria was possibly not the most important cause of death in children under 5 years of age (U5) in Zambia, and that maternal and child health interventions were possibly not the most important cause of the decline in U5 deaths, I checked the data in global burden of disease (GBD) Compare, the data visualization hub on the Institute of Health Metrics and Evaluation website.^2^ The information I obtained (in under an hour—such is the power of GBD Compare) is given in Table 1. In fact, neonatal disorders were the most important causes of U5 deaths in 2010 and 2013 (malaria was most important in 2000), although childhood undernutrition was the most important risk factor. One striking feature in Table 1 is that U5 deaths have been a very high, but decreasing, proportion of total all-age deaths: 39.2% in 2000, 35.4% in 2010, and 33.3% in 2013.

A potentially more informative metric is disability-adjusted life year (DALY) losses. Table 2 shows the DALY losses in 2000, 2010, and 2013 for the causes and risk factors considered in Table 1. This shows a similar ranking of cause and risk factor, and an extremely high, but decreasing, proportion of the overall burden of disease in the U5s: 51.1% in 2000, 47.6% in 2010, and 45.0% in 2013.

What do Tables 1 and 2 tell us about disease priorities? What can Zambia do to reduce U5 deaths and the U5 burden of disease? Obviously, the good work on reducing malarial deaths must be continued. Childhood nutrition is clearly a major issue, including stunting (2,278 U5 deaths in 2013), underweight (5,186), wasting (12,199), suboptimal breastfeeding (4,135 U5 deaths in 2013), and nutritional deficiencies (4,671). Infant- and child-feeding programs should be established, together with nutrition education for mothers and mothers-to-be.^3^

Stunting is strongly associated with unsafe water, sanitation, and hygiene (WASH), especially unsafe sanitation.^4^ Environmental enteropathy is the principal pathway for this association,^5^ which is exacerbated by open defecation (OD). In Zambia, 22% of the rural population—about 2 million people—practices OD.^6^ The elimination of OD is part of the sustainable development goals^7^; and therefore, Zambia needs to get its rural population served with at least basic, and preferably safely-managed, sanitation by 2030.^6^

Lower respiratory-tract infections, particularly pneumonia, exert a high burden of disease and death in the U5s. Pneumococcal conjugate vaccine was launched only in mid-2013,^8^ so it is too early to say how successful this program will be.

Neonatal disorders are very important. Deaths due to neonatal sepsis and other neonatal infections have been rising steadily from 2,121 in 2000 to 2,704 in 2013. This may reflect poor WASH, the difficulty of accessing even basic-level health-care facilities in rural areas, and/or rural mothers not recognizing early symptoms of these diseases. Poor WASH should be addressed, as it is known to adversely affect maternal, infant, and child mortality.^9^ There also needs to be improved rural health care and targeted health/hygiene education for mothers and mothers-to-be.

Diarrheal diseases caused significantly fewer deaths in 2013 than in 2000. This parallels the decrease in unsafe water, unsafe sanitation, and unsafe hand hygiene, which must be sustained. The most dramatic decrease, nearly 60%, was seen in the number of human immunodeficiency virus, acquired immune deficiency syndrome, and tuberculosis deaths during 2010–2013. This truly excellent performance needs, of course, to continue.

As highlighted in this letter, an initial broad-brush approach using GBD Compare (or similar tools) is likely to produce good guidance on health priorities, especially in rural areas and periurban slums. Targeted detail can then follow.

## Figures and Tables

**Table 1:** Number of U5 deaths (both sexes) in Zambia due to selected causes and risk factors in 2000, 2010, and 2013

Cause or risk factor	No. of U5 deaths in 2000 (% of all U5 deaths in 2000)	No. of U5 deaths in 2010 (% of all U5 deaths in 2010)	No. of U5 deaths in 2013 (% of all U5 deaths in 2013)
Causes			
All causes	67,662 (39.2*)	54,485 (35.4*)	48,721 (33.1*)
Malaria	14,234 (21.0)	8,717 (16.0)	8,621 (17.7)
Diarrheal diseases	9,860 (14.6)	5,800 (10.6)	4,963 (10.2)
Neonatal disorders	9,369 (13.9)	9,651 (17.8)	9,838 (20.2)
Lower respiratory-tract infections	9,188 (13.6)	7,492 (13.7)	7,127 (14.6)
HIV/AIDS and TB	7,939 (11.7)	6,421 (11.8)	2,665 (5.5)
Nutritional deficiencies	5,570 (8.2)	4,962 (9.1)	4,671 (9.6)
Risk factors			
Childhood undernutrition	20,555 (30.4)	14,986 (27.5)	13,047 (26.8)
Suboptimal breastfeeding	6,602 (9.8)	4,410 (8.1)	4,135 (8.5)
Unsafe water, unsafe sanitation, and unsafe hygiene†	9,688 (14.3)	5,821 (10.7)	5,032 (10.3)

HIV/AIDS = human immunodeficiency virus/acquired immune deficiency syndrome; TB = tuberculosis; U5 = under 5 years of age.

* The percentage given for ‘all causes’ is all U5 deaths as a percentage of total all-age deaths in the respective year.

† No handwashing with soap.

Source: Institute for Health Metrics and Evaluation.^2^

**Table 2:** Burden of disease in DALY losses in children under 5 years of age (U5) (both sexes) in Zambia due to selected causes and risk factors in 2000, 2010, and 2013

Cause or risk factor	U5 DALY loss in 2000 (years) (% of total U5 DALY loss in 2000)	U5 DALY loss in 2010 (years) (% of total U5 DALY loss in 2010)	U5 DALY loss in 2013 (years) (% of total U5 DALY loss in 2013)
Causes			
All causes	5,885,122 (51.1*)	4,786,355 (47.6*)	4,301,746 (45.0*)
Malaria	1,221,667 (20.8)	749,013 (15.6)	740,896 (17.2)
Diarrheal diseases	860,812 (14.6)	516,510 (10.8)	445,213 (10.3)
Neonatal disorders	811,008 (13.8)	838,295 (17.6)	856,255 (19.9)
Lower respiratory-tract infections	787,506 (13.4)	643,064 (13.4)	611,667 (14.2)
HIV/AIDS and TB	682,070 (11.6)	554,061 (11.6)	231,269 (5.4)
Nutritional deficiencies	504,245 (8.6)	461,896 (9.6)	440,260 (10.2)
Risk factors			
Childhood undernutrition	1,778,100 (30.2)	1,306,887 (27.3)	1,141,755 (26.5)
Suboptimal breastfeeding	571,556 (9.7)	383,387 (8.0)	360,069 (8.4)
Unsafe water, unsafe sanitation, and unsafe hygiene†	845,543 (14.4)	517,576 (10.8)	450,291 (10.5)

DALY = disability-adjusted life year; HIV/AIDS = human immunodeficiency virus/acquired immune deficiency syndrome; TB = tuberculosis.

* The percentage given for ‘all causes’ is the total U5 DALY losses as a percentage of total all-age DALY losses in the respective year.

† No handwashing with soap.

Source: Institute for Health Metrics and Evaluation.^2^
